# B-Cell Response during Protozoan Parasite Infections

**DOI:** 10.1155/2012/362131

**Published:** 2012-01-23

**Authors:** María C. Amezcua Vesely, Daniela A. Bermejo, Carolina L. Montes, Eva V. Acosta-Rodríguez, Adriana Gruppi

**Affiliations:** Centro de Investigaciones en Bioquímica Clínica e Inmunología (CIBICI-CONICET), Departamento de Bioquímica Clínica, Facultad de Ciencias Químicas, Universidad Nacional de Córdoba, Haya de la Torre y Medina Allende, Ciudad Universitaria, 5000 Córdoba, Argentina

## Abstract

In this review, we discuss how protozoan parasites alter immature and mature B cell compartment. B1 and marginal zone (MZ) B cells, considered innate like B cells, are activated during protozoan parasite infections, and they generate short lived plasma cells providing a prompt antibody source. In addition, protozoan infections induce massive B cell response with polyclonal activation that leads to hypergammaglobulnemia with serum antibodies specific for the parasites and self and/or non related antigens. To protect themselves, the parasites have evolved unique ways to evade B cell immune responses inducing apoptosis of MZ and conventional mature B cells. As a consequence of the parasite induced-apoptosis, the early IgM response and an already establish humoral immunity are affected during the protozoan parasite infection. Moreover, some trypanosomatides trigger bone marrow immature B cell apoptosis, influencing the generation of new mature B cells. Simultaneously with their ability to release antibodies, B cells produce cytokines/quemokines that influence the characteristic of cellular immune response and consequently the progression of parasite infections.

## 1. B Cells Can Play Protective and Pathogenic Roles in Protozoan Infections

Host resistance in protozoan infections is dependent on both innate and acquired cell-mediated immune responses. In addition, several studies have implicated B cells and antibodies (Abs) in host survival and protozoan parasite clearance [1–3]. B cells can function as Ab-producing cells but they can also modulate immune responses through critical Ab-independent mechanisms that include secretion of cytokines and chemokines as well as antigen presentation [4–6]. Furthermore, B cells can directly modulate dendritic cells and T-cell subsets, and, consequently, they can influence adaptive immunity and the progression of the infection [7]. Accordingly, in protozoan infections B cells may play a protective and a pathological role. In malaria and trypanosome infections, Abs appear to play a famajor role in immunity. In *Trypanosoma cruzi* and *T. brucei gambiense *infections, Ab-dependent cytotoxic reactions against the parasite have been reported [8]. Several studies demonstrated that Abs are responsible for the survival of susceptible animals in the initial phase of *T. cruzi* infection and for the maintenance of low levels of parasitemia in the chronic phase [9, 10]. Although Abs were shown to be responsible for clearing the African trypanosomes from the blood of infected animals, recent evidence suggests that the survival time of infected mice does not necessarily correlate with the ability of the animal to produce trypanosome-specific antibody. In general, the parasite-specific immune response mounted during protozoan infections is insufficient to completely eradicate the pathogen, allowing chronic infection.

B cells do not only play protective roles in protozoan infections. In fact, they are required for the development of Th2 cell response and, consequently, for the susceptibility to infection with *Leishmania major *[11]. BALB/c uMT mice infected with *L. major *LV39 mount a Th1 response and present restricted lesion development and contained parasite replication. Adoptive transfer of B cells from BALB/c mice in B-cell-deficient BALB/c uMT mice before infection restores susceptibility to *L. major *LV39 and Th2 cell development in resistant mice.

## 2. B-Cell Development

**Figure 1:**
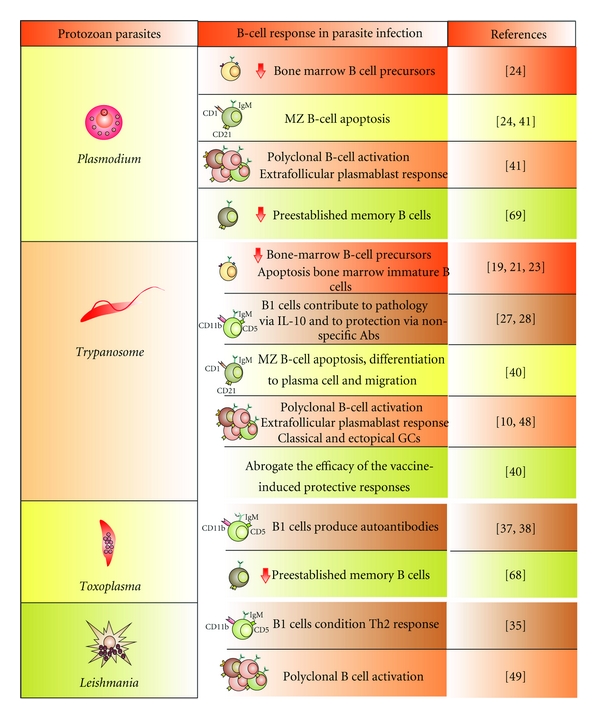
Protozoan parasites affect the different B-cell compartments. MZ: marginal zone B cells, GCs: germinal centers.

Given the role for B cells in conditioning the progression of protozoan infections, it is important to understand the kinetics and regulation of the whole B-cell cycle from the development to the differentiation into mature and memory B cells and plasma cells. The humoral immune response has been shown to be two branched providing an innate-like response (involving B1 and marginal zone (MZ) B cells) and an adaptive immune response (involving conventional B2 cells). In the adult, B cells are generated in the bone marrow (BM) and migrate to the periphery at the transitional B-cell stage, when they are still short lived and functionally immature [12, 13]. Conventional B2-cell development occurs via a series of BM stromal cell-facilitated processes that begin within the hematopoietic stem cell pool and proceed in hierarchical steps of lineage commitment [14]. B lymphopoiesis yields several developmental stages of pre-pro-B, pro-B, pre-B, and, eventually, immature B cells, which show a high expression of the IgM form of the antigen receptor and low or no expression of the IgD maturation marker. To complete their development, immature B cells migrate through the periphery; however, only 10% reach the spleen as transitional B cells of the T1 type [15]. In the spleen, transitional B cells develop into conventional and MZ B cells [16]. B1 cells are efficiently generated in fetal life and during the first few weeks after birth. The fetal liver is an efficient source of B1 cells [17]; however, it is not the only one as a recent study identified a B1-cell precursor in adult BM [18]. Interestingly, protozoan parasite infections can affect the different compartments of B-cell development (summarized in Figure 1), influencing the generation of new mature B cells or their survival and, consequently, the cellular immune response.

## 3. Protozoan Parasite Infections Affect BM B-Cell Development

BM is the main hematopoietic organ of an adult organism and is able to provide cells of immune systems rapidly in cases of infection. Immature B-cell reduction in BM during an infection would limit the Ab source cells and favour parasite replication and chronicity, so the identification of the mechanisms ruling B-cell depletion represents an important challenge in biomedical research.

We have reported that *T. cruzi* infection induces a marked loss of immature B cells in the BM and also compromises recently emigrated B cells in the periphery [19]. The depletion of immature BM B cells was associated with an increased rate of apoptosis, and we established that *T. cruzi* trypomastigotes failed to directly induce immature B-cell apoptosis. We proved that this cell death process occurs in a Fas/FasL-independent fashion but depends on the presence of CD11b^+^ myeloid cells that secrete a product of the cyclooxygenase pathway that depletes immature B cells [19].

In addition, BM is compromised in other protozoan parasite infections. In fact, infections with *Neospora caninum* [20] and *T. brucei* [21] also cause a general decrease in BM cells. Recently, the *T. brucei* infection upshot on B lymphopoiesis has been examined using a C57BL/6 mouse *T. brucei* AnTat 1.1E infection model [22]. Using this model, Bockstal et al. [23] observed that the number of hematopoietic stem cells was minimally affected, but BM B lymphopoiesis was severely affected in *T. brucei*-infected mice, starting with the common lymphoid progenitor fraction. The pre-pro-B-cell population showed a 50% reduction by day 20 after infection, while the subsequent B-cell maturation stage, that is, the pro-B, pre-B, and immature B-cell populations reached more than 95% depletion by day 10 after infection and failed to recover throughout the further course of infection. In *T. brucei* infection, mice do not present increased apoptosis of BM B-cell precursors nor alteration in the expression of B-cell-development-specific transcription factors like Icaros, PU.1, EBF and E2A and the IL-7. However, *T. brucei*-infected mice show a reduction in BM CXCL12 levels [23], indicating that during early *T. brucei* infections, B-cell precursors prematurely migrate out of the BM as a result of the initiation of inflammation. Similarly, CXCL12 decreased production by BM cells was determined in *Plasmodium chabaudi* infection [24]. Furthermore, the significant reduction in CXCL12 expression in the BM of 10 days *P. chaubaudi*-infected mice correlates with a reduction in B-cell precursor cells. At days 20 and 30 of infection, a significant recovery in CXCL12 expression in BM is detected, coinciding with a slow recovery of B lymphopoiesis.

## 4. B1 and MZ B-Cell Response in Protozoan Parasite Infections

Among the mature B cells, MZ and B1 B cells appear to be evolutionarily selected and maintained to facilitate prompt Ab responses. Due to this, they provide a bridge between the innate and the adaptive arms of the antipathogen immune response. B1 cells, distinguished from B2 cells by their phenotype (B220^low^ CD5^+/-^ CD11b^+^) and anatomic location and functional properties, are the dominant population of B cells in the pleural and peritoneal cavities, but represent only a small fraction of splenic B cells [25]. B1 cells produce most of the natural serum IgM and much of the gut IgA and express a BCR repertoire that is enriched for highly polyspecific receptors with low affinities to a broad range of antigens [26]. Despite the fact that B1 cells are very efficient in the control of bacterial and viral infections, they apparently do not play a role in the control of protozoan parasite replication. Indeed, BALB/c Xid mice carrying an X-linked mutation (that prevents B1 cell development) infected with *T. cruzi* display poor B-cell responses to the infection, accompanied by low levels of specific and nonspecific immunoglobulins in the serum [27]. Surprisingly, Xid mice infected with *T. cruzi* were able to control parasitemia and did not show the wasting syndrome observed in wild-type mice. In addition, they developed almost no pathology early in the chronic phase. The resistance of these mice to experimental Chagas disease was associated with the absence of IL-10-secreting B1 cells and high levels of IFN-gamma [28]. These results suggested that B1 cells play a pathological rather than protective role in Chagas' disease. Additionally, in *T. cruzi* infection, we observed a disappearance of peritoneal B1 cells, due to an enhanced differentiation into a particular type of plasma cells, the “Mott-like cells” [29]. Nevertheless, the specific role of these cells in the experimental Chagas disease has not been elucidated yet; their association with autoimmune manifestations in CD22-deficient mice [30] and lupus [31, 32] suggests that these cells may be involved in the autoimmune responses observed in *T. cruzi* infection. We and others have reported that the peritoneal B-cell response observed in *T. cruzi* infection is almost not specific for the invading pathogen [29, 33]. However, these “sticky” antibodies could unspecifically bind to parasites providing protection.

B1 cells are not only implicated on Ab secretion; in fact, they may modulate T-cell response. In this sense, O'Garra et al. [34] have reported that B1 cells secrete large amounts of IL-10 and, consequently, can contribute to the susceptibility of BALB/c mice to *L. major *infection by skewing the T-helper cell network towards a Th2 phenotype. In this way, it has been observed that *L. major *infection of B-cell-defective BALB/c Xid mice induces a less severe disease compared to wild-type control mice [35]. Another report indicates that the behavior of *L. major*-infected Xid mice can be explained more in relation to the high endogenous IFN-gamma production than to the lack of B1 cells. Indeed, B1-cell-depleted irradiated mice showed similar or even worse disease progression compared to control BALB/c mice [36].

B1 cells would also be implicated in the pathogenesis of toxoplasmosis through the production of Abs against the heat shock protein 70 of *T. gondii* that also recognize mice HSP70 [37]. These Abs seem to have a pathogenic role in toxoplasmosis as their injection in *T. gondii*-infected mice clearly increases the number of parasites in mice brain [38]. Moreover, IL-10 produced by B1 cells could, in turn, favor *T. gondii* survival. Then, fine tune regulation of the exacerbated Th1 response by IL-10 is important during *T. gondii* infection.

MZ B cells are also considered innate-like cells that can be induced to differentiate into short-lived plasma cells in the absence of BCR ligation. Splenic MZ B cells can be distinguished from the other splenic B cells by CD24^high^, IgM^high^, IgD^high^, CD23^−^ expression, as well as by their higher expression of CD21. It is known that these B cells mediate humoral immune responses against blood-borne type 2 T-independent antigens [39] but their role in parasite infection has been scarcely studied. Induction of a T-independent anti-trypanosome IgM response has been shown to be a crucial factor in *T. brucei* parasite elimination [1]. Even when increased splenic cellularity occurs after *T. brucei* infection, a significant reduction of splenic IgM+ MZ B-cell numbers takes place right after the first week of infection [40]. The infection-associated disappearance of the MZ B cells from the spleen could be explained by two independent mechanisms, namely, cell differentiation and/or cell death. Supporting the first possibility is the observation that the rapid disappearance of MZ B cells coincided with the temporary accumulation of IgM+ plasma cells. The analysis of MZ B cells that remain in the spleen in the days following the clearance of the first peak of parasitemia revealed that these cells upregulated Annexin V expression. In addition, these cells exhibit caspase 3 gene expression as well as the conversion of procaspase 3 into the cleaved 12 kD and 17 kD caspase 3 activation products suggesting the induction of trypanosomiasis-associated apoptosis in the splenic MZ B-cell population [40]. As in African trypanosomes, *P. chabaudi chabaudi* infection also caused a severe depletion of MZ B cells in the spleen [41] and this loss is mainly the result of the highly increased rate of apoptosis [24]. As MZ B cells serve as an important source for T-cell independently generated IgM+ plasma cells during early stages of infection, apoptosis induction of MZ B cells can be used by parasites as strategy to avoid early IgM protective response and, consequently, prolong their survival.

## 5. Protozoan Infections Induce Massive B-Cell Response with Polyclonal Activation of Splenic B Cells

Whereas the BM of some protozoan-infected mice suffers from a strong B-lineage-cell depletion, the spleens show a marked cellular hyperplasia as a consequence of an intense B-cell response. A detailed analysis of splenic B-cell response was performed in experimental Chagas disease and malaria [10, 41]. An extrafollicular Ab response, in mice infected with *T. cruzi, *is evident a few days after infection and reached a peak after 18 days of infection. This extended kinetics of the extrafollicular response could be characteristic of infections caused by blood circulating protozoan parasites since in *P. chabaudi chabaudi* infection extrafollicular plasmablasts are visible from day 4, and by day 10 they are unconventionally sited in the periarteriolar region of the white pulp. In this region, in both *T. cruzi* and *Plasmodium* infection, extrafollicular plasmablasts form clusters occupying part of the area normally filled by T cells. The kinetics of the appearance of GCs during *T. cruzi* and *Plasmodium* infection are similar to those observed after immunization with classical haptenated proteins, where GCs are visible within the 8 days of immunization [42]. In addition, we detected functional (Ab producing) GCs in atypical sites. The GCs in the spleens of *T. cruzi*-infected mice persisted for at least 32 days resembling the kinetics of the response seen in *P. chabaudi* [41] and *L. amazonensis* [43] infections. We observed that even though *T. cruzi* infection induces early, persistent, and massive extrafollicular and follicular plasmablast responses together with classical and ectopic GCs, infected mice have a delayed parasite-specific Ab response. A key finding of our study [10] is that, while an important amount of Abs is rapidly secreted during infection, antigen specific antibodies were not detected until the third week of infection.

The consequence of the massive extrafollicular and follicular B-cell response is the polyclonal B-cell activation that leads to hypergammaglobulinemia with serum Abs specific for the parasite and self- and/or nonrelated Ags [44–46]. In leishmaniasis, hypergammaglobulinemia was described in both *L. major*-susceptible and -resistant mouse strains. *T. congolense* infection also results in a strong production of non-parasite-specific Abs characterized by the predominance of IgG2a- and IgG2b isotypes [47]. All the mouse strains infected with *T. congolense* present a marked increase in splenic B cells resulting in a nonspecific polyclonal activation of lymphocytes that affects primarily B cells. In strains of *T. congolense* mice which survived longest, that is, C57B1/6J and AKR/A, the increase in splenic B cells is less marked.

Different roles are proposed for polyclonal B-cell activation, which can be crucial for early host defense by contributing with Abs specific for a spectrum of conserved structures present in the pathogens. Additionally, polyclonal activation can be a mechanism triggered by microorganisms to escape the host-specific immune response by diluting pathogen-specific Abs while increasing irrelevant antibodies. Accordingly, recently it has been reported that C57Bl/6 mice, resistant to *T. cruzi* infection, had improved parasite-specific humoral responses that were associated with decreased polyclonal B-cell activation. In the context of parasite infection, Bryan et al. [48] study shows that Th2 cytokine responses were associated with amplified polyclonal B-cell activation and diminished specific humoral immunity. This report demonstrate, that polyclonal B-cell activation during acute experimental Chagas disease is not a generalized response and suggests that the nature of humoral immunity during *T. cruzi* infection contributes to host susceptibility. In leishmaniasis visceral, at early times after infection, there is a marked B-cell expansion in the draining lymph nodes of the site of the infection, which persists throughout infection. As early as day 7 after infection, polyclonal antibodies (TNP, OVA, chromatin) were observed in infected mice and the levels appeared comparable to the specific antileishmania response. Although B-cell-deficient JhD BALB/c mice are relatively resistant to infection, neither B-cell-derived IL-10 nor B-cell antigen presentation appears to be primarily responsible for the elevated parasitemia. Interestingly, passive transfer and reconstitution of JhD BALB/c with secretory immunoglobulins (IgM or IgG; specific or nonspecific immune complexes) results in increased susceptibility to *L. infantum* infection [49].

Another potential deleterious role for polyclonal activation is that it could potentially turn on anti-self-responses and lead to autoimmune manifestations during chronic infections. IgG autoantibodies to brain antigens are increased in *P. falciparum*-infected patients and correlate with disease severity in African children [50]. Autoreactive Abs against endocardium and nerves can be detected in mice and humans infected with *T. cruzi* [51, 52] and are thought to be responsible for much of the Chagas' disease pathological damage. Recently, we reported that BAFF-BAFF-R signaling in *T. cruzi* infection partially controls polyclonal B-cell response but not parasite-specific class-switched primary effectors B cells. BAFF (TNF superfamily B lymphocyte stimulator), a crucial factor for the survival of peripheral B cells [53–55] associated to the development of autoimmune disorders [56], is produced early and persists throughout the infection with *T. cruzi*. By BAFF blockade we observed that this cytokine mediates the mature B-cell response and the production of non-parasite-specific IgM and IgG and influences the development of antinuclear IgG [57].

In addition, polyclonal B-cell activation can be responsible for maintenance of memory B-cell responses because of the continuous, unrestricted stimulation of memory B cells whose Ab production may be sustained in the absence of the antigens binding-specific BCR [58].

## 6. B Cells Influence the Characteristic of Cellular Immune Response Because They Act as APC and Cytokine/Chemokine Producers

Besides being the precursors of the Ab-secreting cells, B cells are committed to do other immune functions such as Ag presentation to T cells or cytokine/chemokine production. It has been widely studied that CD8^+^ CTL are important for protective *T. cruzi *immunity [59, 60] but generally they are not induced by soluble protein vaccines. However, a mechanism known as cross-priming has been described whereby certain professional APC can induce CD8^+^ T-cell responses after the uptake of exogenous Ag [61]. Hoft et al. [62] have demonstrated that the APC functions of B cells may be important for the induction of optimal vaccine-induced responses in mice immunized with a mix of CpG and *T. cruzi *transsialidase, an enzyme involved in parasite infectivity. They also demonstrate that mice deficient in B cells (uMT mice) fails to induce protective immunity when they were immunized with CpG and *T. cruzi *transsialidase. This failure of uMT mice to be protected was associated with the absence of *T. cruzi *transsialidase-specific CD8^+^ T cell response, suggesting that B cells may be important for the cross-priming of CD8^+^ CTL. In addition, it has been reported that *T. cruzi*-infected B-cell-deficient mice have reduced numbers of CD8^+^ splenic T cells and impaired generation of central or effector splenic memory T cells [63]. *T. gondii*-infected C57BL/6 mice develop a robust and uncontrolled Th1 response, and it has been reported that *T. gondii*-primed B cells, but not naive B cells, were able to increase IFN gamma production by splenic T cells *in vivo*. The mechanisms involved may be linked to the presence of membrane-bound TNF on B-cell surface [64].

 The fine tune regulation of migratory cells during infection is an important event in which chemokines and their receptors play a leading role. Different reports have demonstrated that the chemokine receptor CCR5 plays a role in systemic protection and cardiac inflammation during *T. cruzi* infection [65, 66]. CCR5^+^ cells migrate to both mucosal and systemic sites in response to the chemokines CCL3 (MIP-1a), CCL4 (MIP-1b), and CCL5 (RANTES). In line with these reports, Sullivan et al. [67] showed that neutralization of CCL5 in CCR5^−/−^ *T. cruzi*-immune mice results in decreased levels of *T. cruzi*-specific B cell responses and decreased mucosal protection in these mice. They also showed that CCL5 produced by B cells acts in an autocrine manner to increase B cell proliferation and total IgM secretion.

## 7. Established Memory B Cell Response Is Affected by Protozoan Parasite Infections

A hallmark of adaptive immunity is the ability to generate humoral immunological memory by which memory B cells could respond more rapidly and robustly to re-exposure to a new infection. Interestingly, it has been reported that *T. brucei* infection is capable of abrogating the efficacy of the vaccine-induced protective responses against non-related pathogens such as *B. pertussis* [40]. In the same line, Strickland and Sayles [68] showed that *T. gondii* infected mice which were immunized with SRBC had a depression not only in the primary, but also in the secondary humoral immune response, since they showed less IgM and IgG splenic Ab-secreting cells than non-infected control mice. All the data discussed in the present review indicate that protozoan parasites not only affect the development of the cells involved in Ab production [69] but also affect an already established humoral response against other pathogens. Then, the identification of mechanisms able to improve B cell response and, consequently, parasite control will also be beneficial to avoid the deterioration of a memory response to other pathogens.

##  Conflict of Interests

The authors have declared that no competing interests exist.
